# How Long Do the Dead Survive on the Road? Carcass Persistence Probability and Implications for Road-Kill Monitoring Surveys

**DOI:** 10.1371/journal.pone.0025383

**Published:** 2011-09-27

**Authors:** Sara M. Santos, Filipe Carvalho, António Mira

**Affiliations:** 1 Conservation Biology Unit, Department of Biology, University of Évora, Évora, Portugal; 2 Mediterranean Ecosystems and Landscapes Unit, Institute of Mediterranean Agrarian and Environmental Sciences, University of Évora, Évora, Portugal; University of Western Ontario, Canada

## Abstract

**Background:**

Road mortality is probably the best-known and visible impact of roads upon wildlife. Although several factors influence road-kill counts, carcass persistence time is considered the most important determinant underlying underestimates of road mortality. The present study aims to describe and model carcass persistence variability on the road for different taxonomic groups under different environmental conditions throughout the year; and also to assess the effect of sampling frequency on the relative variation in road-kill estimates registered within a survey.

**Methodology/Principal Findings:**

Daily surveys of road-killed vertebrates were conducted over one year along four road sections with different traffic volumes. Survival analysis was then used to i) describe carcass persistence timings for overall and for specific animal groups; ii) assess optimal sampling designs according to research objectives; and iii) model the influence of road, animal and weather factors on carcass persistence probabilities. Most animal carcasses persisted on the road for the first day only, with some groups disappearing at very high rates. The advisable periodicity of road monitoring that minimizes bias in road mortality estimates is daily monitoring for bats (in the morning) and lizards (in the afternoon), daily monitoring for toads, small birds, small mammals, snakes, salamanders, and lagomorphs; 1 day-interval (alternate days) for large birds, birds of prey, hedgehogs, and freshwater turtles; and 2 day-interval for carnivores. Multiple factors influenced the persistence probabilities of vertebrate carcasses on the road. Overall, the persistence was much lower for small animals, on roads with lower traffic volumes, for carcasses located on road lanes, and during humid conditions and high temperatures during the wet season and dry seasons, respectively.

**Conclusion/Significance:**

The guidance given here on monitoring frequencies is particularly relevant to provide conservation and transportation agencies with accurate numbers of road-kills, realistic mitigation measures, and detailed designs for road monitoring programs.

## Introduction

Roads can exert severe impacts upon the long-term viability of animal populations [1], [2], either through direct killings that decrease the number of individuals (road mortality), or through habitat loss and fragmentation, and barrier effects increasing isolation of populations [3]–[5]. Road mortality is one of the best known and visible impacts of roads on animal populations, with millions of individuals from a wide range of taxonomic groups being killed every year [1], [4]. The need for effective mitigation measures to minimize impacts of existing and future roads on wildlife populations [6]–[9] has thus lead to an increasing body of research relating the spatial patterns of road-kills with both ecological and road features [6], [10]–[13]. These studies rely primarily on estimates of road mortality, which are often based on a particular sampling scheme designed for a particular species, thus raising many questions regarding their accuracy and utility for comparative purposes and for guiding monitoring and mitigation plans targeting multiple species.

Several factors have been referred to affect the accuracy of road mortality estimates, including the rate at which the carcasses decompose, the time interval between the occurrence of mortality and road monitoring, the number of vehicles that pass over the carcass, the visibility of carcasses, the abundance and diversity of scavengers, the weather, and the accuracy and precision of the search method [14]–[18]. A common result of studies concerning persistence of carcasses in the field is that carcasses of small animals disappear sooner, usually within the first day of experiments, although some refer to non-road habitats [14], [16], [19]. Some animal carcasses are removed by scavengers, such as corvids, domestic cats, polecats and foxes [15], [16], [20] preventing detection in further surveys. We found only one study that discussed the effect of traffic on persistence. Heavy traffic decreases the access of scavengers to carcasses on the road [15], thus increasing carcass persistence. On the other hand, weather can influence counting numbers in several ways: poor visibility (e.g., fog) and heavy rain or wet conditions increase persistence rates of carcasses on the road by decreasing scavenger activity [15], [18], [21]. Also, a count survey on foot is more effective than on a car vehicle, particularly for smaller animals [15], [17], although it is more costly in terms of time and manpower. Among the factors that influence the accuracy of estimates, the time of carcass persistence, defined as the time each animal carcass remained on the road, has been considered the most important factor inducing bias in road mortality estimates [18].

Although one can easily predict that carcass persistence should be longer for larger animals, and thus that road mortality estimates across different sized species may vary with the frequency of monitoring surveys, few studies have analyzed how carcass persistence probability throughout time may affect road mortality estimates across a large number of taxonomic groups and under different environmental conditions [14], [15], [22], [23]. Moreover, most studies focusing on this subject have been based upon short duration experiences (one-two seasons) using captive-reared bird chicks [14], [23]. The variability in the rate at which carcasses persist on the road in different situations, throughout the year, must be considered when planning road-kill monitoring surveys so that these data can be used as a reliable indicator of mortality [14], [18]. In addition, an assessment of the effect of sampling frequency on the number of road-kills registered in a survey can be of high practical value. Establishing the frequency of road-kill surveys that produce higher accurate estimates at low cost is a major goal in most projects. The implementation of high-cost mitigation measures of new road projects often require monitoring schemes on the effectiveness of those measures, while environmental impact assessment studies must include the design of a monitoring program with a detailed description of the methods and periodicity of controls [1]. The accuracy of road surveys, and the factors influencing the number of road carcasses effectively counted, is a critical point of discussion when striving to provide accurate estimates of wildlife collision rates to conservation and transportation agencies.

This study addressed this issue by using daily surveys of road-killed vertebrates and survival analysis to describe carcass persistence along four sections of roads with different characteristics. Specifically, we aimed to answer the following questions:

1. How long may an animal carcass persist on a road after being hit by a vehicle?
2. How does carcass persistence vary between different taxonomic groups?
3. What is the periodicity of road mortality surveys that minimizes losses through undetected carcass removal?
4. How does carcass persistence changes with road and animal characteristics? And with weather conditions?

In the present work “carcass disappearance” is defined as a carcass that is no longer available for detection from a moving vehicle, meaning that either it becomes unrecognizable for identification (at least to the studied taxonomic groups level), or is absent from the road. This allowed for the calculation of the number of days each road-killed animal remained on the road (persistence time). Overall, we expect that the analysis of different survival curves should differ greatly among taxonomic groups, allowing their classification based on the median expected persistence time, which in turn should be greatly explained by characteristics of road and animals, and by weather conditions. We further expect that our study may provide particularly useful insights regarding the numerical consequences of choosing different time intervals or accuracy targets for road monitoring studies.

## Results

### Global persistence time of animal carcasses on the road

Initially, the persistence time of 4447 animal road-kills were analyzed. Small birds and salamanders were the most abundant groups, accounting for 45% and 18.9% of the casualties, respectively. Large birds and freshwater turtles were among the least represented, comprising just 1.1% and 0.5% of the data sample, respectively. The remaining 8 groups accounted each for somewhere between 9.3% (toads) and 2.0% (bats) of the sample (Table 1).

**Table 1:** Summary of results for persistence estimates for each taxonomic group and the “all taxa” data set (N: sample size; Median (95% CI): median persistence probabilities and corresponding 95% confidence intervals obtained with a Kaplan-Meier estimator; MPT: maximum persistence time recorded (in days); S(t = 1), S(t = 2), S(t = 7): estimate of persistence probability for 1-day, 2-day and 7-day intervals obtained with a Kaplan-Meier estimator).

Taxonomic group	N	Median (95% CI)	MPT (days)	S(t = 1)	S(t = 2)	S(t = 7)
Toads	409	1 (1-1)	12	0.267	0.100	0.010
Salamanders	833	1 (1-1)	15	0.455	0.228	0.016
Lizards	107	1 (1-1)	4	0.056	0.019	0.000
Snakes	146	1 (1-1)	14	0.397	0.212	0.034
Freshwater turtles	22	3 (2-5)	51	0.818	0.591	0.182
Small birds	1990	1 (1-1)	63	0.366	0.203	0.032
Large birds	46	4 (2-6)	51	0.717	0.609	0.283
Birds of prey	110	6 (4-9)	94	0.745	0.673	0.445
Bats	82	1 (1-1)	5	0.146	0.037	0.000
Small mammals	270	1 (1-1)	16	0.389	0.241	0.030
Lagomorphs	208	2 (1-2)	25	0.505	0.351	0.077
Hedgehogs	106	4.5 (3-7)	106	0.774	0.632	0.377
Carnivores	92	9 (5-19)	158	0.804	0.706	0.543
**GLOBAL**	**4447**	**1 (1-1)**	**144**	**0.407**	**0.241**	**0.063**

Most animal carcasses persisted on the road for only one day (or less), after being killed by a vehicle. According to predictions, maximum persistence time varied among different taxonomic groups, ranging from 4 days (for lizards) to 158 days (among carnivores; Table 1).

**Figure 1:**
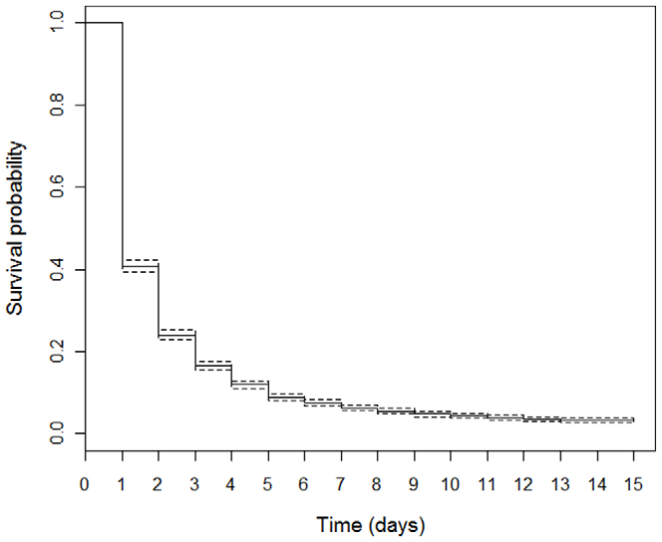
Overall survival curve for the “all *taxa*” data set (Kaplan-Meier estimate and corresponding 95% confidence intervals; N = 4447; the length of time axis is limited to a maximum of 15 days).

The median of “all *taxa*” persistence time was one day and corresponded to a 0.5 probability of persistence. This probability decreased to 0.241 on the second day (i.e. 76% of road-killed animals did not persist on the road for longer than two days). These values indicate a low global persistence probability; with a substantial drop beyond the first day (Fig. 1; Table 1).

### Differences in persistence time between taxonomic groups

**Figure 2:**
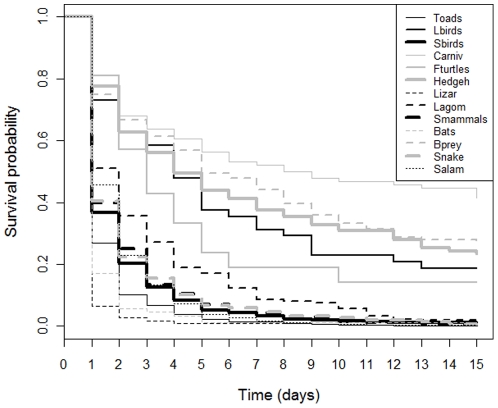
Separate survival curves for the 13 taxonomic groups; Toads: toads (including frogs), Lbirds: large birds (more than 200 g, and excluding raptors), Sbirds: small birds (less than 200 g), Carniv: carnivores; Fturtles: freshwater turtles, Hedgeh: hedgehogs, Lizar: lizards, Lagom: lagomorphs, Smammals: small mammals, Bats: bats, Bprey: birds of prey (diurnal and nocturnal), Snake: snakes, Salam: salamanders (including newts); (Kaplan-Meier estimates; N = 4447; the length of time axis is limited to a maximum of 15 days).

Table 1 and Fig. 2 summarize the persistence time and simple survival functions for the taxonomic groups considered. Median persistence time varied between one (for seven of the 13 *taxa*) and nine days (for carnivores). All seven taxonomic groups with lower median time (toads, salamanders, lizards, snakes, small birds, bats, and small mammals) presented persistence probabilities lower than 0.50 after the first 24 h, meaning that, after that time, fewer than 50% of road-killed animals from these *taxa* were still on the road. Survival curves provided the classification of each taxonomic group according to their persistence time (median and maximum values). Very short persistence time (<1 day) was characteristic of lizards and bats (the lowest values observed); and short time (1 to 2 days) was registered for toads, salamanders, snakes, small birds, small mammals, and lagomorphs. Freshwater turtles, large birds, birds of prey, and hedgehogs had medium persistence time (3 to 6 days); and carnivores had the longest persistence time on the road (>7 days; see Fig. S1, S2, and S3 in Supporting Information).

### Influence of monitoring periodicity on road-kill numbers

To discuss the implications of varying time frequencies in monitoring studies, three scenarios of sampling frequency were defined: 7-day, 2-day and 1-day intervals. In addition, two threshold persistence probabilities also were defined: 0.50 and 0.70. These values were chosen to represent two different possible monitoring goals (and distinct financial budgets), and were assumed to represent, respectively, that roughly 50% and 70% of road-kills are still recorded by a monitoring program. Thus, for a scenario of weekly road monitoring (7-day interval), carnivores and birds of prey were the only groups with nearly a 0.50 probability of persisting on the road. All other *taxa* exhibited lower probabilities, most of them less than 0.10. Within a scenario of road monitoring every 3 days (2 day-intervals between surveys), more than a 0.50 probability of persistence existed for freshwater turtles, large birds, birds of prey, hedgehogs and carnivores. For this monitoring frequency, all other groups had less than a 0.25 persistence probability. Considering the scenario of monitoring on alternate days (1 day-intervals), carnivores, freshwater turtles and hedgehogs had more than a 0.75 persistence probability; birds of prey, large birds and lagomorphs a probability between 0.50 and 0.75; salamanders, snakes, small mammals, small birds and toads a probability between 0.25 and 0.50; and bats and lizards less than a 0.25 probability of persistence (Table 1).

According to the previously defined monitoring goals and budgets, considering 50% to be an acceptable persistence probability (assuming a lower financial budget), the periodicity of road monitoring that minimizes bias on road mortality estimates is: daily monitoring for lizards (in the afternoon period) and bats (in the early morning), toads, small birds, small mammals, snakes, and salamanders; a one-day interval for lagomorphs; a two-day interval for freshwater turtles; a three-day interval for large birds and hedgehogs; a 4-day interval for birds of prey; and an 8-day interval for carnivores (Fig. 2; Fig. S1, S2, and S3). To achieve a goal of 70% persistence (assuming a greater financial budget), the corresponding periodicity of road monitoring would be: daily monitoring for lizards (in the afternoon) and bats (in the early morning); daily monitoring for toads, small birds, small mammals, snakes, salamanders, and lagomorphs; alternate days for large birds, birds of prey, hedgehogs, and freshwater turtles; and every three days for carnivores (Fig. 2; Fig. S1, S2, and S3). For a general monitoring program directed at capturing all vertebrate groups, daily monitoring would be required to attain at least a 50% persistence probability across all *taxa* (Fig. 1). Specific periods of the day are justified for monitoring lizards and bats, because they exhibited very short persistence time (<1 day). Accordingly, monitoring surveys should be conducted as close as possible to their activity (or mortality) periods: lizards are killed during daylight hours, while bats are killed overnight.

### Influence of road, animal and weather on persistence time

#### “All *taxa*”

The Cox model for the “all *taxa*” data set (stratified by taxonomic group) was highly significant (P<0.001), and explained 9.9% of the variance in carcass persistence time. According to this model, carcass persistence probability was lower for animals weighing less than 20 g when compared to higher body masses; in roads with very low traffic (less than 1000 vehicles/day); in paved lanes when compared to unpaved shoulders; and during periods with higher proportion of rainy days in the wet season (November–April), and with higher minimum temperatures in the dry season (May–October; Table 2).

**Table 2:** Multivariate Cox hazards models for the persistence time of “all *taxa*” data set, stratified by taxonomic group (β: coefficients, e^β^: hazards ratio, e^β^ LCI 95%: 95% lower confidence interval for hazards ratio, e^β^ UCI 95%: 95% upper confidence interval for hazards ratio, z: Wald test, P value: significance of Wald test); performance parameters of the model: R^2^ = 0.099; Likelihood test = 464.9, df = 14, P-value<0.0001.

Variables	β	e^β^	e^β^LCI 95%	e^β^UCI 95%	z	P value
bmass (2)	−0.115	0.891	0.818	0.971	−2.627	0.009
bmass (3)	−0.798	0.450	0.368	0.550	−7.809	<0.0001
bmass (4)	−1.559	0.210	0.040	1.103	−1.844	0.065
raindays	0.432	1.541	1.421	1.671	10.491	<0.0001
traffic (2)	0.068	1.071	0.958	1.197	1.211	0.226
traffic (3)	−0.054	0.947	0.853	1.051	−1.022	0.307
traffic (4)	−0.240	0.786	0.698	0.886	−3.946	<0.0001
rposition (2)	0.089	1.093	0.973	1.228	1.495	0.135
rposition (3)	−0.051	0.950	0.881	1.025	−1.330	0.183
rposition (4)	−0.177	0.837	0.725	0.968	−2.401	0.016
season (2)	−0.201	0.818	0.621	1.077	−1.433	0.152
mintemp	−0.001	0.998	0.985	1.012	−0.216	0.829
season (2) * raindays	−0.265	0.767	0.695	0.847	−5.238	<0.0001
season (2) * mintemp	0.064	1.066	1.043	1.090	5.723	<0.0001

As an example of interpretation of the effect of body mass on persistence probabilities, based on estimates of the hazards ratio (or risk ratio, RR), the carcass of a <20 g animal was about half as likely to persist as a 100 to 1000 g carcass (RR respectively 0.89 and 0.45), and animals weighing more than 1000 g were almost 80% more likely to persist (RR = 0.21) (Table 2).

#### Taxonomic groups

Overall, Cox models were significant for most taxonomic groups considered, explaining 6.4 to 66.8% of variance in carcass persistence time (bats and freshwater turtles, respectively), with an average value of 23.8% (n = 12 models; Tables 3, 4, 5, 6, and 7). It was not possible to build a significant model for lizards, since none of the available explanatory variables lowered the Akaike's Information Criterion (AIC; [24]) comparing with the null model. For bats, the best explanatory variable was not significant itself, nor did it contribute for a significant model. Despite this, the model for bats is presented (Tables 3 and 4).

**Table 3:** Summary of performance parameters of Cox proportional hazards models for the 12 taxonomic groups analyzed (R^2^: explained variance, LL test: Likelihood test, df: degrees of freedom, P-value: significance of Likelihood test).

Taxonomic group	R^2^	LL test	df	P-value
Bats	0.064	5.39	2	0.067
Toads	0.174	78.01	6	<0.0001
Salamanders	0.159	144.70	6	<0.0001
Small mammals	0.108	30.92	4	<0.0001
Snakes	0.166	26.45	4	<0.0001
Small birds	0.141	301.70	13	<0.0001
Lagomorphs	0.066	14.12	5	0.015
Freshwater turtles	0.668	24.23	3	<0.0001
Large birds	0.463	28.64	5	<0.0001
Hedgehogs	0.322	41.25	4	<0.0001
Birds of prey	0.093	10.76	3	0.013
Carnivores	0.427	51.17	8	<0.0001

**Table 4:** Multivariate Cox hazards models for Bats, Toads, Salamanders, and Small mammals (β: coefficients, e^β^: hazards ratio, e^β^ LCI 95%: 95% lower confidence interval for hazards ratio, e^β^ UCI 95%: 95% upper confidence interval for hazards ratio, z: Wald test, P-value: significance of Wald test, for variables definition, see table 8).

Variables	β	e^β^	e^β^LCI 95%	e^β^UCI 95%	z	P-value
**Bats**						
rposition (2)	−0.588	0.555	0.270	1.141	−1.602	0.109
rposition (3)	0.218	1.244	0.775	1.994	0.905	0.366
**Toads**						
bmass	−0.007	0.993	0.990	0.996	−5.005	<0.0001
raindays	0.310	1.364	1.050	1.772	2.323	0.020
mintemp	0.065	1.067	1.032	1.105	3.745	<0.001
traffic (2)	0.317	1.373	0.961	1.961	1.740	0.082
traffic (3)	0.202	1.224	0.905	1.658	1.311	0.190
traffic 4)	−0.094	0.910	0.650	1.275	−0.546	0.585
**Salamanders**						
raindays	0.793	2.209	1.775	2.751	7.091	<0.0001
season (2)	−2.445	0.087	0.031	0.240	−4.706	<0.0001
bmass	−0.042	0.959	0.926	0.993	−2.325	0.020
rainamount	0.075	1.078	0.999	1.163	1.932	0.053
mintemp	0.030	1.030	0.995	1.066	1.690	0.091
season (2) * mintemp	0.198	1.219	1.117	1.329	4.467	<0.0001
**Small mammals**						
raindays	0.509	1.663	1.327	2.085	4.418	<0.0001
bmass	−0.229	0.795	0.686	0.922	−3.042	0.002
rainamount	−0.234	0.791	0.659	0.951	−2.497	0.012
mintemp	0.024	1.025	0.999	1.051	1.892	0.058

**Table 5:** Multivariate Cox hazards models for Snakes, Small birds, and Lagomorphs (β: coefficients, e^β^: hazards ratio, e^β^ LCI 95%: 95% lower confidence interval for hazards ratio, e^β^ UCI 95%: 95% upper confidence interval for hazards ratio, z: Wald test, P-value: significance of Wald test, for variables definition, see table 8).

Variables	β	e^β^	e^β^LCI 95%	e^β^UCI 95%	z	P-value
**Snakes**						
raindays	0.408	1.504	1.069	2.116	2.343	0.019
rainamount	−0.489	0.613	0.463	0.811	−3.431	<0.001
mintemp	0.089	1.093	1.032	1.159	3.017	0.002
bcondition (2)	−0.308	0.735	0.499	1.082	−1.561	0.118
**Small birds**						
raindays	0.485	1.624	1.446	1.824	8.202	<0.0001
rainamount	−0.313	0.731	0.666	0.802	−6.609	<0.0001
bmass	−0.220	0.802	0.726	0.887	−4.283	<0.0001
traffic (2)	0.107	1.113	0.915	1.355	1.074	0.283
traffic (3)	−0.089	0.915	0.771	1.086	−1.015	0.310
traffic (4)	−0.294	0.745	0.619	0.898	−3.099	0.002
rposition (2)	0.188	1.207	0.993	1.468	1.888	0.059
rposition (3)	−0.050	0.951	0.856	1.057	−0.932	0.351
rposition (4)	−0.052	0.949	0.795	1.134	−0.571	0.568
season (2)	0.068	1.071	0.741	1.547	0.364	0.715
Mintemp	0.004	1.004	0.985	1.023	0.383	0.701
season (2) * raindays	−0.204	0.815	0.702	0.947	−2.676	0.007
season (2) * mintemp	0.045	1.046	1.015	1.078	2.953	0.003
**Lagomorphs**						
traffic (2)	−0.783	0.457	0.159	1.317	−1.450	0.147
traffic (3)	−1.103	0.332	0.120	0.915	−2.132	0.033
traffic (4)	−1.305	0.271	0.091	0.805	−2.352	0.019
raindays	0.348	1.417	1.049	1.914	2.272	0.023
rainamount	−0.312	0.732	0.547	0.979	−2.100	0.036

**Table 6:** Multivariate Cox hazards models for Freshwater turtles, Large birds, and Hedgehogs (β: coefficients, e^β^: hazards ratio, e^β^ LCI 95%: 95% lower confidence interval for hazards ratio, e^β^ UCI 95%: 95% upper confidence interval for hazards ratio, z: Wald test, P-value: significance of Wald test, for variables definition, see table 8).

Variables	β	e^β^	e^β^LCI 95%	e^β^UCI 95%	z	P-value
**Freshwater turtles**						
rposition (3)	−3.377	0.034	0.006	0.178	−4.004	<0.0001
rposition (4)	2.755	15.718	1.366	180.787	2.211	0.027
mintemp	0.266	1.305	1.072	1.588	2.656	0.008
**Large birds**						
season (2)	1.452	4.274	2.095	8.718	3.993	<0.0001
rposition (2)	0.792	2.208	0.705	6.913	1.361	0.174
rposition (3)	0.918	2.504	1.195	5.250	2.431	0.015
rposition (4)	−1.267	0.282	0.099	0.799	−2.382	0.017
bmass	−0.005	0.995	0.991	0.998	−2.706	0.007
**Hedgehogs**						
raindays	1.958	7.087	3.063	16.394	4.576	<0.0001
season (2)	1.770	5.870	2.596	13.268	4.253	<0.0001
rainamount	−0.823	0.439	0.292	0.661	−3.945	<0.0001
season (2) * raindays	−1.080	0.340	0.128	0.902	−2.167	0.030

**Table 7:** Multivariate Cox hazards models for Birds of prey and Carnivores (β: coefficients, e^β^: hazards ratio, e^β^ LCI 95%: 95% lower confidence interval for hazards ratio, e^β^ UCI 95%: 95% upper confidence interval for hazards ratio, z: Wald test, P-value: significance of Wald test, for variables definition, see table 8).

Variables	β	e^β^	e^β^LCI 95%	e^β^UCI 95%	z	P-value
**Birds of prey**						
season (2)	1.163	3.200	1.316	7.779	2.566	0.010
rainamount	−0.459	0.632	0.414	0.964	−2.131	0.033
mintemp	−0.086	0.917	0.847	0.993	−2.124	0.034
**Carnivores**						
raindays	1.998	7.375	3.714	14.643	5.710	<0.0001
rainamount	−1.356	0.258	0.153	0.434	−5.108	<0.0001
season (2)	0.794	2.212	0.994	4.924	1.945	0.052
rposition (2)	−0.713	0.490	0.153	1.567	−1.202	0.229
rposition (3)	−0.604	0.546	0.327	0.913	−2.309	0.021
rposition (4)	−1.438	0.237	0.092	0.615	−2.959	0.003
bcondition(2)	−0.652	0.521	0.320	0.847	−2.631	0.008
mintemp	−0.085	0.919	0.853	0.989	−2.238	0.025

Results of Cox models revealed that the weather related variables (“season”, “raindays”, “rainamount”, and “mintemp”; see Table 8 for variable details) were the most important, each of which being retained in six to eight models (out of the total of 12 models), covering *taxa* with short to long persistence time (Tables 4, 5, 6, and 7). Higher proportion of “raindays” decreased carcass persistence probability for toads, salamanders, small mammals, snakes, lagomorphs, and carnivores (Tables 4, 5, and 7). However, a larger “rainamount” only decreased the persistence probability for salamanders (with borderline significance; P = 0.053), while increasing persistence for small mammals, snakes, small birds, lagomorphs, hedgehogs, birds of prey, and carnivores (Tables 4, 5, 6, and 7).

**Table 8:** List of explanatory variables, their definition, and values.

Variable	Definition	Values
road	Identification of the road where road-kill was registered	1: M529/M370; 2: N4; 3: N114
traffic^a^	Class of road traffic volume	1: <1 000 vehicles/day (M370); 2: 1 000 4 000 vehicles/day (M529); 3: 4 000–10 000 vehicles/day (N4A, N4B, N114A); 4: >10 000 vehicles/day (N114B)
rposition	Position of the carcass on the road	1: lane; 2: center; 3: paved shoulder; 4: unpaved shoulder
bcondition	Integrity status of the carcass	1: full carcass; 2: remains
Bmass^b^^,^ c	Mean body mass of species (or taxonomic group) according to bibliographic references (see text)	3.3–7300 g (1: <20 g; 2: 20–100 g; 3: 100–1000 g; 4: >1000 g)
blength	Mean body length of species (or taxonomic group) according to bibliographic references (see text)	3.7–151.7 cm
season	Period of the year in which the carcass was initially found	1: Nov–Apr (wet season); 2: May–Oct (dry season)
raindays^d^	Proportion of days with rainfall during the time the animal remained on the road (number of days with rain/number of days the animal remained on the road)	0–1
rainamount^e^	Rainfall abundance during the time the animal remained on the road (total amount of rainfall/number of days the animal remained on the road)	0–41.9 mm/day
meantemp	Daily mean temperature during the time the animal remained on the road	2.2–30.2°C
maxtemp	Daily maximum temperature during the time the animal remained on the road	7.1–40.8°C
mintemp	Daily minimum temperature during the time the animal remained on the road	−3.6–21°C

a values of traffic intensity for each road segment after E.P.E. (2005) and our own data (António Mira, unpublished data).

b logarithmic transformation for small birds, small mammals and birds of prey;

c body mass was considered continuous for taxonomic groups data, and categorical for the “all *taxa*” data set;

d arcsine transformation for all taxonomic groups;

e logarithmic transformation for all taxonomic groups.

The influence of temperature also varied between groups: higher “mintemp” decreased the persistence probability for snakes, small mammals (with borderline significance; P = 0.058), and freshwater turtles; while higher “mintemp” increased the persistence for birds of prey and carnivores (Tables 4, 5, 6, and 7). The persistence probability of large birds, birds of prey and carnivores decreased during the dry season (Tables 6 and 7). Some interactions were significant, particularly season with proportion of rainy days for small birds and hedgehogs, and season with minimum temperature for small birds and salamanders (interpreted as for the “all *taxa*” model; Tables 4, 5, and 6).

Two variables not pertaining to weather were also retained commonly in built multivariate Cox models: “bmass” and “rposition”. Road-killed animals with higher body masses showed higher persistence probabilities for salamanders, small mammals, and small and large birds (Tables 4, 5, and 6). The carcasses of small birds located at the center of the road were less likely to persist than those found on the lanes. Persistence probability also decreased on paved shoulders (when compared with lanes) for large birds, and increased for freshwater turtles and carnivores (Tables 6 and 7). On the other hand, the persistence probability on lanes relatively to unpaved shoulders was higher for freshwater turtles, and lower for large birds and carnivores (Tables 6 and 7). For bats, the position of carcasses on the road seemed to have some importance with persistence probability increasing in road center and decreasing in paved shoulders relative to lanes, though neither of the class coefficients was significant (Table 4).

“Traffic” and “bcondition” were selected only in three and two models, respectively (although some classes in these variables were not significant). Accordingly, persistence increased along road segments with 4 000 to 10 000 vehicles/day (when compared with the lowest class) for lagomorphs; and along segments with the greatest traffic (i.e., more than 10 000 vehicles/day when compared with the lowest class) among small birds and lagomorphs. Non-intact carcasses of carnivores and snakes were more likely to persist on the road, though this result is non-significant in the latter group (Tables 5 and 7).

## Discussion

### Carcass persistence time on the road

Most animal carcasses on roads are quickly dismembered by passing vehicles, eaten or removed by scavengers and predators, or reduced to skeletons by ants and other decomposers [16], [17], [23]. In the present study, most carcasses remained on the road for the first day only, with some groups disappearing at high rates over this first day. Our results are in accordance with several published short-term experiences, some of them applied to road persistence time, and some to other habitat types, most involving bird carcasses. One of the few studies that has tested persistence time in open fields across several species, identified a similar range of loss values (24 to 98% of carcasses disappeared within 24 h), which were explained through scavenger activity alone [16]. The remaining literature on birds, includes a wider variety of results, from persistence time similar to our data for birds with variable sizes (10 to 62% loss in 24 h; [25], [26]), to lower loss estimates (5 to 40% loss; [27]–[30]). Other authors have estimated lower persistence time for both small birds [14], [19], [20], [31] and medium-sized birds [20], [23], [30], [32] than those reported here. There also are persistence estimates published for amphibians and snakes on roads, which are respectively similar to and lower than the values documented in the present study [17], [21], [23]. As described, most available studies (road and non-road habitats) generated lower probabilities of carcass persistence over time relatively to our data. A possible explanation for this could be differences in experimental design, including: shorter observation time (e.g. a few weeks or just two seasons); frequent use of dead animals that are placed artificially (rather than wild animals actually killed by vehicles in that place); and small sample sizes. Globally, these differences might lead to higher disappearance rates. Inter-study differences might also be explained on the basis of different regional conditions, such as different climates, habitats, communities of scavengers and predators, or types of roads (see discussion below).

### Classification of taxonomic groups according to persistence time

Persistence time was highly variable among the different taxonomic groups analyzed in our study, from very short (less than one day) to relatively long periods (more than seven days). Besides differences in body size, certain species traits may make them more or less likely to persist on the road. For example, animals that are covered by fur, spines or scales are more resistant to vehicles passing over them than amphibians [15], [21], [33], though some species of amphibian (e.g. *Salamandra salamandra*) may remain longer on the road due to their tough skin and unpalatability [21], [34]. On the other hand, some species may be removed more frequently than others by scavengers and predators [15], or even persons. ANTWORTH and collaborators [23] suggest that long, linear snakes are more readily detected and recognized on the road as food than small birds. In addition, small carcasses may have a wider range of potential scavengers than larger species do, and may be more rapidly destroyed by invertebrates, like ants [15], [29]. Another source of carcass removal can be related to “human clean-up” of roads. During the course of this study, there was only occasional removal of very large carcasses by road crews. However, a few situations during field work suggest that, occasionally, persons remove carcasses from the road: intact lagomorphs and partridges recently road-killed (for eating), and carnivores and birds of prey (for taxidermy and scientific studies; authors, pers. observ.).

### Monitoring frequency in road-kill studies

A weekly schedule (or wider spaced interval) for road mortality monitoring studies should be avoided, even when larger species with higher persistence probabilities are the target. If the study is not too constrained by its budget, we suggest monitoring with 2-day intervals for carnivores; alternate days for large birds, birds of prey, hedgehogs, and freshwater turtles; and daily for all other groups. According to our results, studies focusing on the broad vertebrate community should be based on daily monitoring. Longer intervals between surveys will underestimate the number of road-kills for most species, especially smaller animals (nearly 80% of road casualties; [13]), creating a bias towards lower kill rates for such animals. For species of small size, which include many threatened *taxa* (like European bats), the required monitoring frequencies are much higher than those typically found in many published studies. This may have resulted in road-kills underestimation in many cases [35], [36], which in turn may have prevented effective mitigation. efforts regarding animal road mortality. Although there have been studies incorporating daily frequencies of road-kill sampling of general taxonomic groups [10], [37], [38], a large number of surveys have been conducted once weekly [18], [28], [39], [40] or even bi-weekly [13], [41], [42]. The present study shows that sampling intervals longer than one day can seriously underestimate road-kill numbers, especially for smaller animals with observed losses of almost 60% overall, and of more than 85% and 73% for bats and lizards, respectively. STEWART [31] and ERRITZOE et al. [20] both have suggested that, ideally, road-killed small birds should be monitored 2–3 times each day. If we could relate persistence time with classical information theory, such as the Shannon-Nyquist sampling theorem, we would also suggest sampling twice a day for those smaller *taxa* to reach minimal accurate estimates (i.e., assuming that persistence time is an estimate for the fundamental frequency of a “carcass signal”; [43], [44]). However, sampling two times a day for long periods implies very high costs (financial and manpower), and would only be advisable for specific situations.

When surveying only larger animals, like carnivores or owls, a sampling frequency of once every 15 days is commonly found in the literature [6], [11], although even more prolonged intervals have been adopted [45]–[47]. This monitoring frequency can lead to high rates of carcass loss; our own numbers suggest that losses near 60 and 75% could be observed for carnivores and birds of prey, respectively.

### Factors influencing persistence time among vertebrates

#### “All taxa”

Several factors exerted an overall effect on the persistence probability of wildlife carcasses on roads. As expected, carcass persistence probabilities were smaller for small-sized animals, because they are crushed and torn apart faster by the continuous impact of car wheels, and are more easily removed or consumed by scavengers and predators than larger carcasses [15].

Carcasses found along roads with lower versus higher traffic volumes, and located on road lanes versus unpaved shoulders were less likely to persist than their counterparts. Our results regarding road lanes agree with other authors' results for birds [23], [31]. Although one might expect that carcasses located on roads with high traffic volume would have lower persistence probabilities, due to larger number of vehicles passing directly over them, our data revealed opposing results. This might be explained by the additional influence of scavenger and predator activity, removing road-killed animals from roads, particularly from road locations with better access [48]. In fact, lower volumes of traffic allow easier access of avian and mammalian predators to eat or remove dead animals [20]. Moreover, the location of carcasses on road lanes not only makes them more susceptible to being repeatedly damaged by vehicles, it also renders them more visible to avian predators that hunt regularly along roads ([23], [48]; author's pers. observ.).

There are several species that include carrion in their diet. The most common are corvids, birds of prey, and mammalian carnivores; but communities of invertebrate decomposers also are very relevant, due to their abundance and diversity; and hedgehogs and rats also are occasional consumers [20]. The contribution of predators and scavengers to decreasing carcass persistence probability has been well documented [17], [20], [23], [31] and their influence is also suggested by the present work. Several avian predators have been frequently observed eating or carrying road-killed prey in the study area; such scavengers include kites (*Milvus milvus* and *M. migrans*), buzzards (*Buteo buteo*), carrion crows (*Covus corone*), and magpies (*Pica pica*) (author's pers. observ.). Mammalian carnivores were also detected in the vicinity of the studied roads looking for food; these include domestic cats (*Felis catus*), foxes (*Vulpes vulpes*), and Egyptian mongooses (*Herpestes ichneumon*) (António Mira, unpublished data). In addition, several studies report the consumption of road carrion by most of these species [15], [20], [49], further supporting the importance of scavenging in observed road persistence probabilities. There are also some reported cases of carcass removal by regular persons or road crews, although in our study it is incidental and directed to certain species (see text above).

The present study also documented how carcasses experiencing more humid conditions during the wet season and higher temperatures during the dry season exhibit shorter persistence time. Rainy days during the cooler months of the wet season should promote faster carcass dismemberment by vehicles passing over them, comparing with drier and cold days, which facilitate the preservation of animal tissues [17]. Although higher temperatures during the dry season could enhance the rapid desiccation of animal bodies, thereby increasing persistence probabilities [33], it also can promote higher activity rates of microorganisms and invertebrate decomposers (ants, coleoptera, maggots, etc.; [27], [50]), accelerating the disappearance of carcasses. Indeed, ants can transform a recently road-killed small bird into a skeleton in just one hour (F. Carvalho, pers. observ.). Our results are in accordance with those of several other authors, who have claimed that carcass persistence is lower in summer months than in spring or autumn, due to increased temperatures and the diversity of insect communities [50], [51] or scavenger activity [52], [53]. In addition, elevated temperatures during summer increase the formation of volatile and smelly chemicals that can attract scavengers and predators to the carcasses [54]. On the other hand, predator and scavenging activity by vertebrates can increase during the dry season due to the greater energy needs of seasonal offspring and the later abundance of juveniles [55], [56].

#### Taxonomic groups

Regarding the influence of explanatory variables on persistence probabilities among different taxonomic groups, certain seemingly-related variables occasionally exerted opposing effects (i.e., one variable increasing persistence while another variable decreased it). For example, carcass persistence probability among several *taxa* decreased with the proportion of rainy days, but increased with larger amounts of rain. Although ostensibly contradictory, one explanation for this is that, although humid days favor carcass softening and dismemberment, intensive precipitation can reduce predator activity, especially hunting flights by avian predators [52], [53]. On the other hand, the effect of the amount of rain could influence some taxonomic groups differently, as certain species are not among preferred prey. For example, although most amphibians tend to be road-killed on rainy days, salamanders generally are avoided by most predators [57] and thus remain longer on the road than most toads and frogs under this weather conditions.

Although higher temperatures decreased persistence probability among most taxonomic groups, birds of prey and carnivores remained longer on the road under higher temperature conditions. The higher body masses and dimensions of these *taxa* could explain such differences. Larger carcasses are unlikely to be carried away by vertebrate scavengers [15], [30] as few predators in the area can easily remove such weights. The most probable situation is that, after soft body parts have been eaten by scavengers and predators, the high temperatures promote hardening of the remaining body fur or feathered skin, which stay on the road in a desiccated form for prolonged periods of time (author's pers. observ.).

Carcass persistence probabilities were much reduced during rainy days in the wet season for small birds and hedgehogs, and at high temperatures during the dry season for small birds and salamanders. This is in accordance with the “all *taxa*” model and could reflect the specific climatic conditions that favor carcass persistence within each season (as previously discussed) or the seasonal differences in predator activity and abundance in the area.

Once again, differences in persistence probabilities between road shoulders and lanes should reflect different scavenging strategies by avian and mammalian predators. The high probabilities of carnivore carcass persistence on shoulders can be explained by the marked decrease in the amount of damage caused by vehicles passing over them in this part of the road, and the lower expected range of scavengers eating larger species [29], [30]. Conversely, lagomorphs frequently are taken as prey by road scavengers [46], or even by persons, when recently killed (authors, pers. observ.). Feeding on a road-killed carcass poses its own risks for predators (and persons when stopping and getting out of the vehicle), this risk being less along roads with lower traffic intensity. Thus, higher levels of traffic intensity promoted higher persistence probabilities for lagomorphs.

Carnivores and snakes remained longer on the road as body remains (as opposed to intact animals). For snakes, it is commonly observed in the field that intact animals are more frequently carried away by predators, while remains get fixed to the pavement and skin scales persist for a long time ([17]; authors, pers. observ.). For carnivores, this result might be explained by the partial consumption of carcasses by vertebrate scavengers that cannot remove higher weight animals from the road [15], [30].

### Monitoring implications

To obtain realistic and complete pictures of wildlife road mortality, particular roads must be monitored in a systematic way and at regular intervals over at least a year [38]. The present study highlights the numerical consequences of choosing different time intervals for road monitoring studies, focusing, for the first time, on a wide variety of European vertebrate groups in a large-sample study. Our results suggest that, although variable among taxonomic groups, persistence time of vertebrate carcasses on the road are globally short, thus requiring that road mortality estimates should be based on high quality data collected at fine-temporal scales (one day or even shorter intervals). The guidance given here regarding monitoring frequencies is particularly useful for studies aiming the recognition of road-kill patterns, and the identification of road mortality hotspots for impact assessment and validation of mitigation measures. In fact, recent results suggest that distinct frequencies of road monitoring can lead to differences in the spatial location of road mortality hotspots (A. Mira et al. in prep). The application of mitigation measures outside real mortality hotspots may be financially costly and environmentally ineffective in reducing wildlife mortality on roads.

The present study highlights, for the first time, the effects of multiple factors on the persistence of road-killed carcasses from a large and diverse sample of wild vertebrates. Our results also suggest that scavengers and predators may exert a strong influence on carcass persistence probabilities, and that their activity (and thus removal rate) might, in turn, depend upon the aforementioned factors (season, weather, location, and animal group). Moreover, differences in the diversity and abundance of predator communities between different geographic regions should lead to distinct carcass persistence probabilities. The carcass removal by scavengers and predators should be studied further in different regions and landscape contexts because, besides differences in population abundances, scavengers and predators with different sizes, periods of activity or food preferences must affect differently the probabilities of carcass persistence.

The results reported here may have practical implications in designing effective road mortality monitoring surveys. In particular, results showed that the accuracy of road monitoring counts can be greatly improved by adapting monitoring methods and frequencies according to local weather conditions, traffic volume, and size of target species. The yearly schedule of surveys may be adjusted to account for mean expected precipitation and temperatures, so as to account for varying carcass persistence time (e.g., more frequent surveys during rainy months). Also, the timetable of surveys may be attuned to the target *taxa*. However, different weather conditions through the world may affect different species differently, and monitoring frequencies should be adapted. Surveys directed towards smaller species should be of higher concern and must be conducted at higher frequencies, particularly in humid seasons as these conditions generally promote the shortest persistence time on roads. This can be critical under other climate regimes, such as monsoonal rains, where persistence time should be even shorter. Traffic volume is another factor to consider, as less traffic facilitates scavenger access to carcasses. Designing road mortality monitoring surveys should thus require adjustments in sampling efforts according to target groups and local environmental conditions. Besides increasing the accuracy of estimates, the use of standardized multiple species road-kill surveys will allow the comparison of results between studies, and the improvement of mitigation proposals.

We caution that the results from our work are not universal in their application, as the mean body size of some taxonomic groups discussed here may differ in other regions of the world. For instance, most of the European bat species are of small size (under 20 g; [58]) when compared to South American or Australian bats (e.g. several species of flying-foxes can weigh up to 1 000 g; [59]). Also, our work is limited to species under 10 kg weigh, which implies that carnivore results apply to medium-sized predators (a common scenario in most European countries).

Moreover, different weather conditions in distinct geographic regions are likely to influence carcass persistence probabilities. The overall trends reported here must verify in many regions. However, specific carcass removal rates may change, tending to be higher in warmer and/or wet regions. Thus, an adjustment of proposed survey intervals may be needed in regions where weather conditions are markedly different. Even so, we believe that our recommendations are valid in many circumstances, although being particularly useful in Mediterranean areas.

Most studies documenting the negative impact of roads make inferences from the number of road-kills documented during road surveys. We have quantified biases associated with different monitoring frequencies and taxonomic groups under different environmental conditions, highlighting how the numbers counted may not accurately reflect the actual number of road-kills.

## Materials and Methods

### Study area

This study was conducted in southern Portugal (38°32′24″ to 38°47′33″N, 08°13′33″ to −07°55′45″W), in an area between the cities of Montemor-o-Novo (west), Évora (east) and Arraiolos (north). The relief is smooth and undulating, with elevations ranging from *ca.* 150 m to 400 m above sea level. The study area is dominated by the typical Mediterranean forest known as “montado” (50.8%; cork and/or holm oak trees with agro-silvo-pastoral use; [60]) and agricultural areas (44.0%; mainly dry arable land, sparse “montado”, and olive orchards and vineyards). Weather is characteristic of Mediterranean climates with minimum and maximum mean temperatures of 5.8°C and 12.8°C during the winter (January), respectively, and 16.3°C and 30.2°C in the summer (July); annual rainfall averages 609.4 mm (Évora 1971–2000; [61]). Four road sections were selected within this area, with varying traffic volumes, and summing to 37 km in total length. Roads N4 and N114 (12 km and 9.5 km, respectively) are classified as national roads, while M529 and M370 are municipal roads (9 km and 6.5 km, respectively). All roads are two-lanes wide, without central barriers/dividers, except in two road crossings. The national roads have paved and unpaved shoulders; while the municipal roads have unpaved shoulders only. All animals used in the present study were already found dead (road-killed), and therefore an ethic approval is not required. All efforts were made to minimize suffering of animals found still alive after being hit by a vehicle, delivering them as soon as possible to wildlife recovering centers.

### Road-kill survey

From December 2004 to February 2006, the four roads were surveyed daily by vehicle to detect road-killed vertebrates. Surveys began within 2 h of sunrise. The standard road sampling width corresponded to both lanes and shoulders (paved and unpaved). Surveys were conducted by one observer driving 20 km per hour and checking both sides of the road. Whenever a road-killed animal was detected, the species was identified *in loco* (or most accurate taxonomic position for carcass remains), and the geographic coordinate position was registered with a hand-held global position system (GPS) unit with 5 m-accuracy (Garmin eTrex Venture). The body condition of the carcass (full carcass or remains), and the position on the road (center, lanes, paved or unpaved shoulder) were registered. All the carcasses were left in the same position in which they were initially found, and during subsequent surveys their presence was rechecked to determine how long they lasted before disappearing. This allowed for the calculation of the persistence time.

Other explanatory variables were added later: classes of traffic volume for each road section, mean body mass and length of each species, and average meteorological conditions during the period of carcass persistence (proportion of days with rainfall, amount of rainfall, mean daily temperature, minimum daily temperature and maximum daily temperature) (see Table 8).

Traffic intensity values for different segments of the studied roads were obtained from national road reports [62] and from our own data (António Mira, unpublished data). The classes of traffic intensity were defined as in IUELL et al. [1]: roads with 1000 or fewer vehicles/day were considered permeable to most species (M370); roads with 1001 to 4000 vehicles/day were deemed permeable to most species, but avoided by sensitive species (M529); roads with 4001 to 10 000 vehicles/day were considered strong barriers associated with high road mortality (N4 and a section of N114); and roads with more than 10 001 vehicles/day were assumed to be impermeable to most species (one section of N114).

Mean body mass and length for each species were obtained from diverse bibliographic references, and corresponded to estimates made for adults of both sexes [63]–[67].

Meteorological information was obtained from the meteorological weather station located in Mitra/Évora (Geophysics Center of Évora, University of Évora). Two variables concerning rain were defined: “raindays” quantifies the duration of periods of high humidity, and “rainamount” quantifies the amount of rain that falls over a certain time period. Accordingly, prolonged periods with little rain, a common event in the study area, tend to produce relatively small quantities of effective rainfall.

### Data analyzes

The original data were categorized into 13 data matrices, corresponding to the 13 most representative taxonomic groups of road-killed vertebrates: toads (anurans, including frogs; 4 to 125 g weigh); salamanders (urodeles, including newts; 25 to 30 g weigh); lizards (lacertids; 3 to 340 g weigh); snakes (colubrids; 15 to 240 g weigh); freshwater turtles (chelonids; 290 to 300 g weigh); small birds (passeriformes, coraciiformes and piciformes; with 8 to 200 g weigh); large birds (birds weighing between 200 g and 1200 g, excluding birds of prey); birds of prey (diurnal and nocturnal; accipitriforms and strigiforms; 175 to 1100 g weigh); bats (chiropters; 6 to 23 g weigh); small mammals (arvicolids, murids, soricids and talpids; 11 to 300 g weigh); lagomorphs (*Oryctolagus cuniculus* and *Lepus granatensis*; 1100 to 2300 g weigh); hedgehogs (*Erinaceus europaeus*; 850 g weigh); and mammalian carnivores (111 to 7300 g weigh). Global data (including the 13 taxonomic groups) were also analyzed (referred to in the text as “all *taxa*”).

Median carcass persistence was estimated and compared among different taxonomic groups using the Kaplan-Meier estimator [68]. The survival curves and probabilities produced with this method allowed the comparison of overall persistence time among the 13 taxonomic groups.

The influence of explanatory variables (road and animal characteristics, and weather conditions; see Table 8) on the persistence probabilities of carcasses was assessed by means of Cox proportional hazards models [69]. The interpretation of both the survival functions, described by the Kaplan-Meier estimator, and the Cox model correspond, in the present context, to the probability that a carcass remains on the road until a specified time, with low hazard ratios (and negative Cox model coefficients) indicating higher persistence probabilities.

Preliminary screening of variables was undertaken with exploratory plots and simple Cox survival models [70]. Arcsin transformation was applied to “raindays”, and logarithmic transformation to “rainamount” for all data matrices. Logarithmic transformation was applied to “bmass” for the matrices of small birds, small mammals, and birds of prey [71]. Pearson correlations among all pairs of continuous variables (or chi-square tests for categorical variables) were calculated to check for multicollinearity [71]. For pairs of variables exhibiting correlation values greater than 0.7 [72] only the strongest predictor in simple Cox models was used in further analysis.

Multiple Cox survival models were constructed for each data matrix (for the “all *taxa*” data set and each single taxonomic groups), and selection of the best model was based on AIC [24]. Interaction terms among weather variables “season” and “traffic” were considered during model selection. The “all *taxa*” model included the taxonomic group as a stratified variable in order to account for different baseline hazards for each *taxon* [73].

The overall importance of variables in the Cox models was evaluated through the log-likelihood chi-square test, while coefficients for individual variables in the models were evaluated with the Wald test and 95% confidence intervals for the hazard ratios (not including the value of 1.0). Competing models (differences in AIC values less than 4) were evaluated with the chi-square test, and most complex models were retained only if a significant (P<0.05) decrease in AIC was observed. The number of explanatory variables in each model was limited to a number such that a ratio of 7 to 10 cases per variable could be achieved [74]. The proportional hazards assumption was tested using a global chi-square test that compares transformed survival time with scaled Schoenfeld residuals, and plots the variation of coefficients for each variable in the model through time (the Kaplan-Meier transformation was chosen as it tends to spread residuals uniformly across the plot; [68]). Whenever the global chi-square test of proportional hazards assumption was significant (P<0.05), the plots of problematic variables were used to assess whether the degree of temporal deviation from estimated coefficients displayed no major deviation, meaning that the proportional hazards assumption was accepted [68]. In order to evaluate each model's goodness-of-fit, we considered the overall likelihood ratio (LR) test, the proportion of variance explained (R^2^), and the plots of Martingale residuals against explanatory variables [70]. Influential observations were assessed with plots of dfbetas residuals. Interpretation of strength of association between explanatory variables and carcass persistence probabilities was based upon respective hazards ratios, and only significant variable classes (Wald test) included in the models are highlighted in the Results section.

We used the software R version 2.10.1 [75] and the package Survival [76] in all model building procedures.

## Supporting Information

Figure S1 Kaplan-Meier estimates for individual survival functions of Lizards, Bats, Toads, Salamanders, Small birds, and Snakes, showing the persistence probability and 95% confidence intervals (the length of time axis is limited to a maximum of 15 days, when available, to allow comparison between groups).
(TIFF)

Figure S2 Kaplan-Meier estimates for individual survival functions of Small mammals, Lagomorphs, Large birds, Birds of prey, Hedgehogs, and freshwater turtles, showing the persistence probability and 95% confidence intervals (the length of time axis is limited to a maximum of 15 days, when available, to allow comparison between groups).
(TIFF)

Figure S3 Kaplan-Meier estimates for individual survival function of Carnivores showing the persistence probability and 95% confidence intervals (the length of time axis is limited to a maximum of 15 days, when available, to allow comparison between groups).
(TIFF)
