# Supplementary figures and images for: How Long Do the Dead Survive on the Road? Carcass Persistence Probability and Implications for Road-Kill Monitoring Surveys
Source: PLoS One. 2011 Sep 27;6(9):e25383. doi: 10.1371/journal.pone.0025383 (PMC3181337; doi:10.1371/journal.pone.0025383)

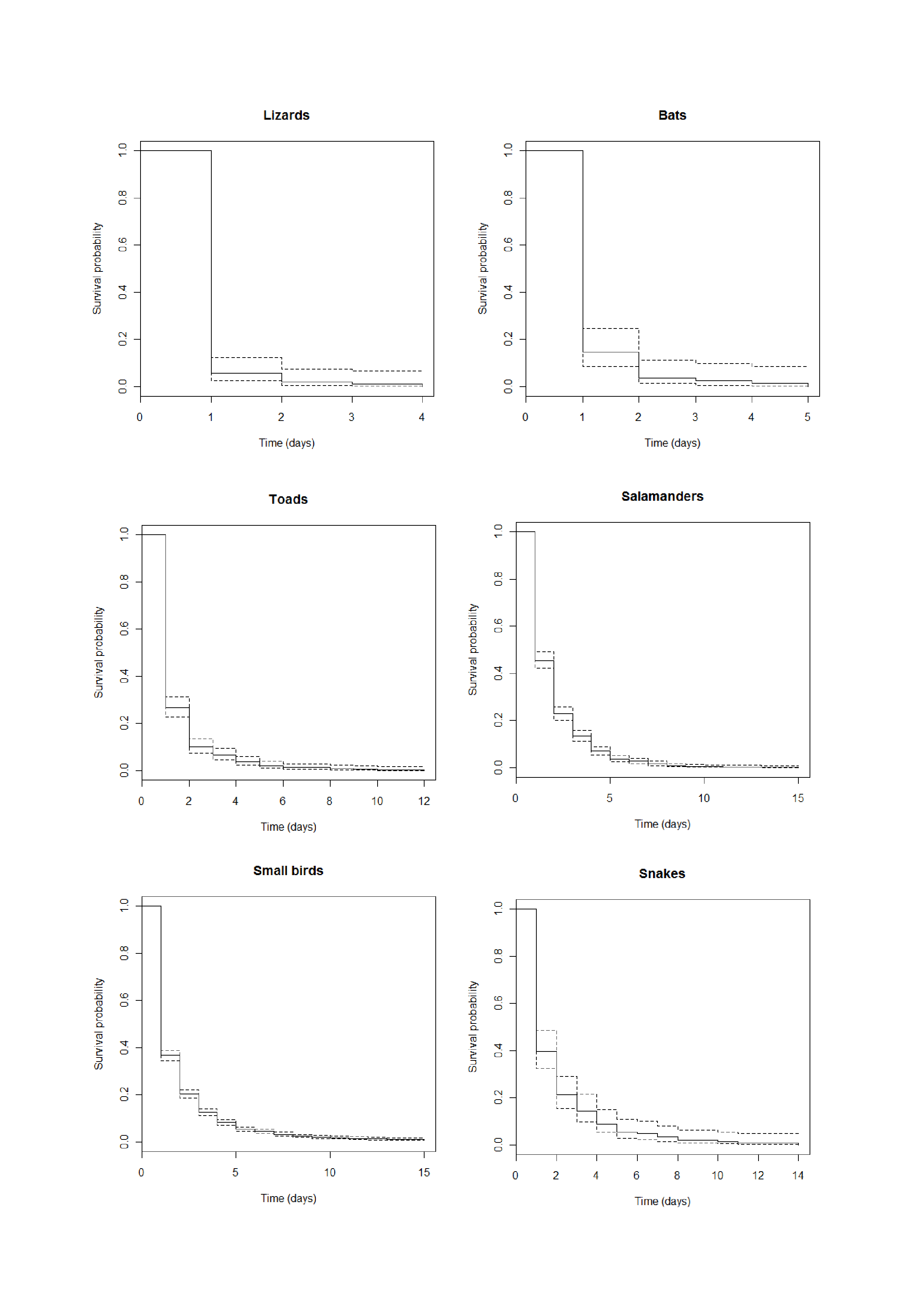

Supplement: Figure S1 — Kaplan-Meier estimates for individual survival functions of Lizards, Bats, Toads, Salamanders, Small birds, and Snakes, showing the persistence probability and 95% confidence intervals (the length of time axis is limited to a maximum of 15 days, when available, to allow comparison between groups). (TIFF) [file pone.0025383.s001.tiff]

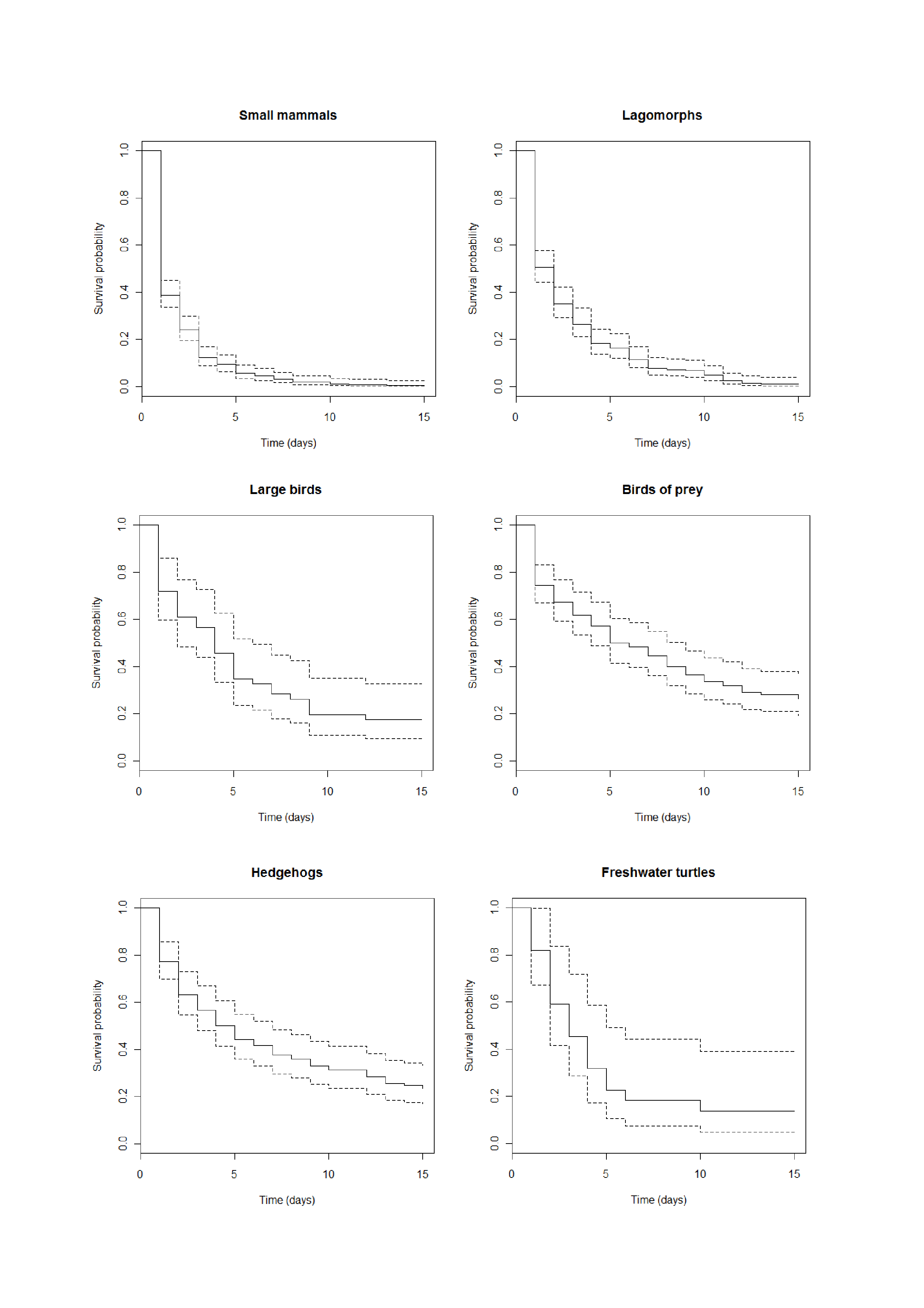

Supplement: Figure S2 — Kaplan-Meier estimates for individual survival functions of Small mammals, Lagomorphs, Large birds, Birds of prey, Hedgehogs, and freshwater turtles, showing the persistence probability and 95% confidence intervals (the length of time axis is limited to a maximum of 15 days, when available, to allow comparison between groups). (TIFF) [file pone.0025383.s002.tiff]

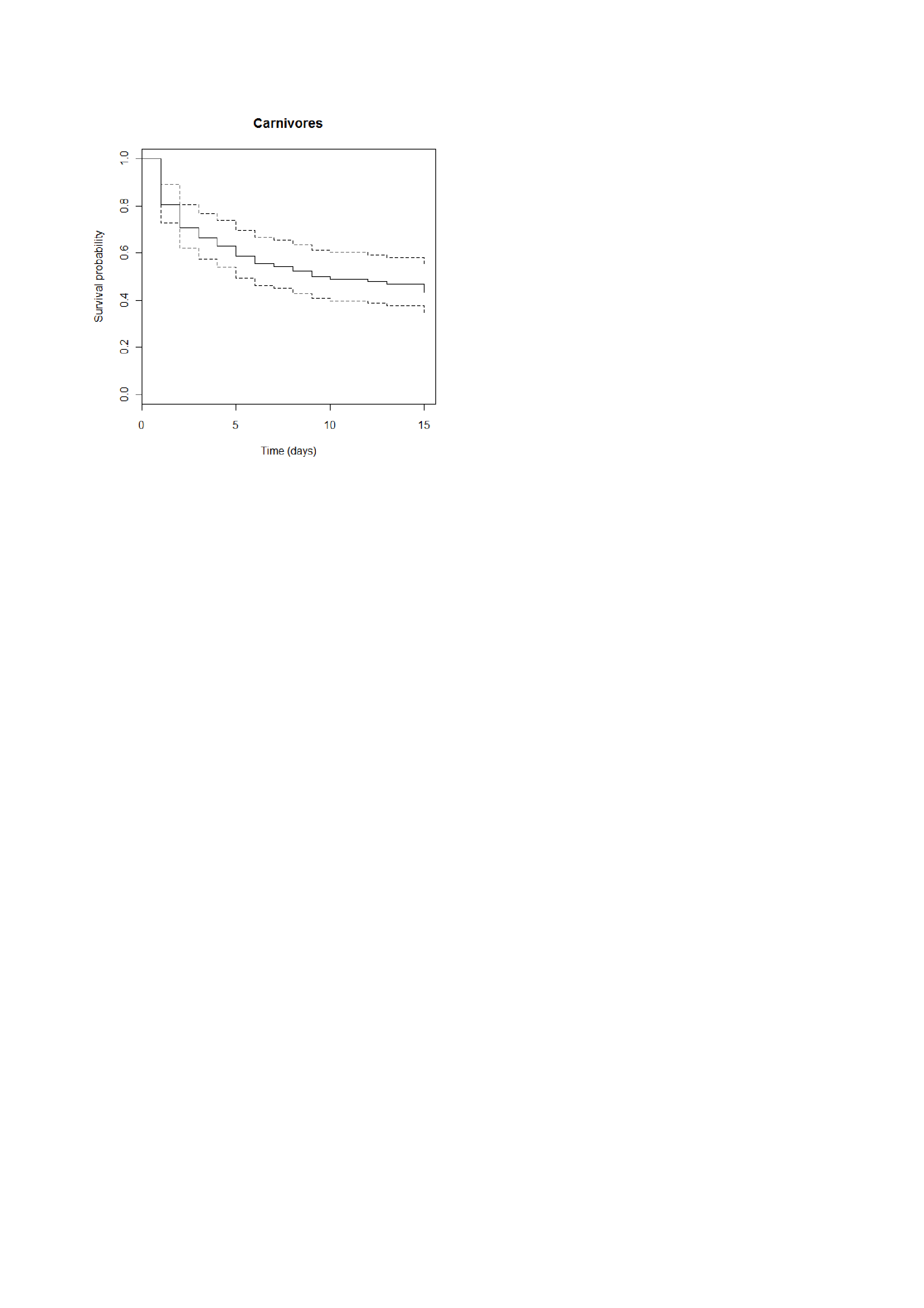

Supplement: Figure S3 — Kaplan-Meier estimates for individual survival function of Carnivores showing the persistence probability and 95% confidence intervals (the length of time axis is limited to a maximum of 15 days, when available, to allow comparison between groups). (TIFF) [file pone.0025383.s003.tiff]
